# What is the risk of subsequent contralateral spontaneous pneumothorax following surgical management of a unilateral pneumothorax?

**DOI:** 10.1186/1749-8090-10-S1-A358

**Published:** 2015-12-16

**Authors:** Naveed Abbas, David Healy

**Affiliations:** 1 Department of Cardiothoracic Surgery St Vincent's University Hospital Elm Park, Dublin, Republic of Ireland

## Background/Introduction

Recurrent spontaneous pneumothorax prompts surgical intervention. Even though the underlying pathology is still not clear, there is an increased risk of spontaneous pneumothorax of the opposite lung. In many cases patients' wish to know the risk of a further event on the opposite side and whether prophylactic surgery would be of benefit.

## Aims/Objectives

Calculate risk of contralateral pneumothorax in patients with recurrent pneumothorax on one side.

## Method

A retrospective review of data from a prospectively recorded database was performed from April 2011 to December 2014. We identified 64 cases of unilateral pneumothorax managed with VATS surgical interventions, after excluding cases secondary to cystic fibrosis. Cases were followed and those developing contralateral spontaneous pneumothorax were recorded.

## Results

A total of 64 cases were performed. The average follow-up was 21 months. In three cases contralateral spontaneous pneumothorax occurred (4.6%). Two cases presented within one month of the first procedure and the third at 8 month. As these had a prior history, surgical intervention was advocated in the three cases.

## Discussion/Conclusion

Contralateral pneumothorax following surgical intervention for spontaneous pneumothorax is not common but does occur. However the frequency is not such that prophylactic intervention is advocated. Routine screening follow-up is unlikely to be rewarding and so counselling and provision of information regarding symptoms that would prompt re-representation is advocated.

